# Molecular response patterns in relapsed/refractory AML patients treated with selinexor and chemotherapy

**DOI:** 10.1007/s00277-022-05075-4

**Published:** 2022-12-28

**Authors:** Piroska Klement, Walter Fiedler, Razif Gabdoulline, Louisa-Kristin Dallmann, Clara Philine Wienecke, Johannes Schiller, Christian Kandziora, Katrin Teich, Bennett Heida, Konstantin Büttner, Maximilian Brandes, Carolin Funke, Martin Wichmann, Basem Othman, Joerg Chromik, Stefanie Amberg, Maxim Kebenko, Vera Schlipfenbacher, Anne Christine Wilke, Franziska Modemann, Melanie Janning, Hubert Serve, Carsten Bokemeyer, Susann Theile, Ute Deppermann, Anne L. Kranich, Arnold Ganser, Felicitas Thol, Michael Heuser

**Affiliations:** 1 Department of Hematology, Hemostasis, Oncology and Stem Cell Transplantation, Hannover Medical School, Carl-Neuberg Str. 1, 30625 Hannover, Germany; 2 Department of Oncology, Hematology and Bone Marrow Transplantation With Section Pneumology, Hubertus Wald University Cancer Center, University Medical Center Hamburg-Eppendorf, Hamburg, Germany; 3 Medical Clinic II, Hematology, Hemostaseology, Medical Oncology, Rheumatology, Infectious Disease, University Hospital Frankfurt, Frankfurt, Germany; 4 GSO Global Clinical Research B.V., Amsterdam, The Netherlands; 5 Mildred Scheel Cancer Career Center, University Cancer Center Hamburg, University Medical Center Hamburg-Eppendorf, Hamburg, Germany; 6 GSO mbH, Hamburg, Germany

**Keywords:** AML, Relapse, Molecular patterns, Selinexor

## Abstract

Relapse in patients with acute myeloid leukemia (AML) is common and is associated with a dismal prognosis. Treatment options are limited and the understanding of molecular response patterns is still challenging. We analyzed the clonal response patterns of 15 patients with relapsed/refractory AML treated with selinexor in a phase II trial (SAIL). DNA was analyzed at three time points and showed a decline of mutated alleles in *FLT3*, *SF3B1*, and *TP53* under SAIL treatment. Overall survival (OS) was similar between patients with declining versus persisting clones. We show an interesting long-term course of a patient who relapsed after allogeneic stem cell transplantation (alloHCT) with SF3B1- and SRSF2-mutated AML and received selinexor as maintenance treatment for 4 years. Measurable residual disease (MRD) remained detectable for 2 weeks after donor lymphocyte infusion (DLI) in this patient and then remained negative under selinexor maintenance treatment. Selinexor was tolerated well and was stopped after 4 years of SAIL treatment. We present an exploratory study and identify subclonal patterns of patients treated with selinexor.

**Supplementary Information:**

The online version contains supplementary material available at 10.1007/s00277-022-05075-4.

## Introduction

Selinexor is an exportin-1 (XPO-1) inhibitor that forces the nuclear retention and functional activation of tumor suppressor proteins, thereby inducing apoptosis in cancer cells [1, 2]. Overexpression of XPO-1 is common in many tumors, including acute myeloid leukemia (AML) [3]. New therapies are particularly needed in relapsed AML, as 10–60% of all AML patients will relapse and have a poor prognosis [4]. Following a promising phase I trial, [5] we conducted a phase II study with selinexor plus cytarabine and idarubicin in patients with relapsed/refractory AML (SAIL) [6]. Forty-two patients with a median age of 59.5 years were enrolled. Due to prolonged aplasia and a rate of febrile neutropenia of 85% and of grade 3/4 diarrhea of 56%, the initial selinexor dose of 40 mg/m^2^ twice weekly for 4 weeks was reduced to 60 mg twice weekly for 3 weeks resulting in a reduction of febrile neutropenia and severe diarrhea to 33% and 40%, respectively. The overall response rate (complete remission (CR), CR with incomplete hematologic recovery (CRi), and MLFS) was 50%. Fifteen patients had a first or second stem cell transplantation (36%). The event-free, relapse-free, and overall survival were 4.9, 17.7, and 8.2 months, respectively. Three of four NPM1-mutated patients responded, but a detailed molecular workup has not been reported. Response to selinexor has been previously associated with t(6;9) resulting in the fusion protein *DEK-NUP214* [7]. To identify molecular predictors of response and survival, we evaluated the molecular profile and time course and correlated them to the clinical outcome of patients treated with selinexor and chemotherapy in the SAIL trial [6].

## Patients, materials, and methods

### Patients and treatment

All 42 patients who were treated in the SAIL trial were eligible to participate in this correlative study. The SAIL trial was a phase II study evaluating selinexor with cytarabine and idarubicin in relapsed or refractory AML patients according to the 2016 World Health Organization criteria [8]. Patients received standard chemotherapy (7 + 3, continuous infusion of cytarabine 100 mg/m^2^ on days 1–7 and idarubicin 10 mg/m^2^ intravenously on days 1, 3, and 5) plus selinexor 40 mg/m^2^ orally twice a week for 4 weeks or 60-mg selinexor absolute twice a week for 4 weeks [6]. The main criterion for inclusion in the present study was availability of DNA from bone marrow or peripheral blood at 3 time points: initial AML diagnosis, screening for the SAIL trial, and first response assessment after cycle 1 (scheduled for day 28 of cycle 1, median after 17 days). Fifteen patients had DNA available and were included in the present analysis. Written informed consent was obtained according to the Declaration of Helsinki and the study was approved by the local ethics committee.

### Cytogenetic and molecular analyses

G- and R-banding analysis was performed centrally in blood or bone marrow samples. DNA was extracted and processed as described previously [9]. A custom TruSight myeloid sequencing panel (Illumina, San Diego, CA) was used to determine mutations associated with myeloid leukemias including 46 genes (Supplementary Table S1). DNA sequencing libraries were prepared from samples (bone marrow *n* = 40, peripheral blood *n* = 8) at diagnosis, at relapse (respectively the start of selinexor), and at follow-up according to the manufacturers’ instructions (Illumina, San Diego, CA) and as described previously [9].

### Error-corrected sequencing for sensitive MRD detection

Sensitive measurable residual disease (MRD) assessment was used to monitor the follow-up samples of the long-term patient for mutations in SF3B1 and SRSF2 (Supplementary Table S2). An amplicon sequencing approach for sensitive detection of SNVs and indels to reduce the sequencing error rate was applied as described before [9, 10]. The Illumina MiSeq reagent kit v3 (600 cycles, San Diego, USA) was used for sequencing and was run on the MiSeq sequencer aiming for a high coverage per sample. This amplicon-based error-corrected sequencing and bioinformatics approach was applied to samples of the long-term patient 6.4, 6.48, 6.56, 6.6, 9.2, and 11.3 years after initial diagnosis.

### Whole-genome amplification

Since ultra-deep sequencing requires a high amount of DNA, some samples had to undergo amplification to increase the DNA amount. The Qiagen REPLI-gMini Kit was used according to the manufacturer’s instructions to amplify genomic DNA [11].

### Bioinformatics and statistical analyses

Bioinformatics analysis of myeloid panel sequencing and of error-corrected sequencing was performed as previously described according to a standardized algorithm for calling single nucleotide variants (SNVs) and small and large insertions/deletions (indels) MRD positive or negative based on the number of read families (RF mode, error-corrected sequencing) or the number of matching forward (R1) and reverse (R2) reads (R1/R2 mode), using the background error of the individual sample to define the limit of detection [9, 10]. Limit of detection (LOD) for SNVs and small indels was defined as an average of the background error plus 3 standard deviations of the background error, where background error is quantified by LVAF (largest non-reference variant allele fraction at all nucleotide positions between the primers of the respective amplicon). For large indels, ≥ 75 supporting (mutated) reads were required to call MRD positive, except for the *NPM1* 4 base pair insertion, where the requirement was ≥ 10 supporting reads.

For NGS-MRD analyses, bioinformatic analysis was performed using a sensitive error-corrected amplicon sequencing approach, which had a sensitivity threshold of 0.015%, to validate identified variants [10, 12].

Molecular response was defined as variant allele frequency (VAF) negativity in the follow-up sample in comparison to the relapse sample, i.e., a mutation found at the time of relapse was no longer detectable in the follow-up sample with a sensitivity of 1%. Molecular non-response was defined as a VAF ≥ 1% at relapse that was still detectable at follow-up.

Overall survival (OS) endpoints, measured from the date of start of selinexor, were death (failure) and alive at last follow-up (censored). Event-free survival (EFS) endpoints, measured from the date of start of selinexor, were relapse (failure), molecular non-response (failure), death in complete remission (CR) (failure), and alive in CR at last follow-up (censored). The Kaplan–Meier method and log-rank tests were used to estimate the distribution of OS and EFS, and to compare differences between survival curves. Categorized variables were considered in univariate analysis for EFS and OS. Comparisons of variables were performed using the Kolmogorov–Smirnov test and the Chi-squared test for categorical variables for exploratory purposes. The two-sided level of significance was set at *P* < 0.05. The statistical analyses were performed with the statistical software package SPSS 26.0 (IBM Corporation, Armonk, NY), statistical program R using packages “survival,” and Microsoft Excel 2021 (Microsoft Corporation, Redmond, WA, USA).

## Results

### Patient characteristics

**Fig. 1:**
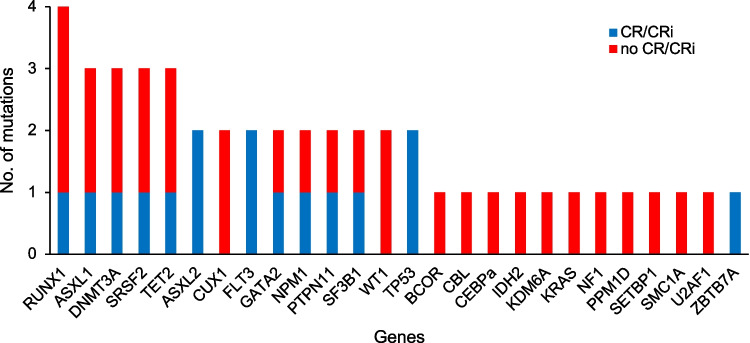
Frequency of genes that were found mutated at the time of SAIL screening and association with CR/CRi

Fifteen (36%) of 42 patients were included for whom DNA was available for the time points at diagnosis, at relapse/refractoriness, and at the first response assessment at the end of the first cycle of selinexor and chemotherapy (Supplementary Fig. S1). Included patients were younger than the excluded patients but did not differ among other clinical variables (Supplementary Table S3), nor for EFS or OS (Supplementary Fig. S2). Baseline characteristics of all 15 patients are listed in Supplementary Table S4. The median age was 49.1 years (range 29–72). Eleven (73%) patients had de novo and four (27%) had secondary/therapy-related AML. Nine patients belong to the favorable or intermediate cytogenetic risk groups, whereas four patients were classified as adverse. The molecular profile showed a predominance of secondary AML-type mutations (Fig. 1).

### Response to selinexor and chemotherapy

All patients received one course of SAIL treatment. Seven (47%) patients achieved morphologic complete remission (CR) or CR with incomplete hematologic recovery (CRi). Clinical characteristics were similarly distributed between CR/CRi patients and all other patients (Supplementary Table S5). When comparing the molecular characteristics of patients achieving CR/CRi and all other patients, no trend to achieve CR/CRi could be observed (Supplementary Table S6).

Clonal evolution of patient-specific mutations was evaluated from diagnosis to relapse/refractoriness to post SAIL treatment (Supplementary Fig. S3). Clones with mutations in *FLT3* (*FLT3*-TKD = 1, *FLT3*-ITD = 1), *SF3B1*, and *TP53* declined under SAIL treatment, whereas clones with mutations in *CUX1*, *GATA2*, *TET2*, *BCOR*, *DNMT3A*, *RAD21*, *ASXL1*, *SRSF2*, *RUNX1*, *NPM1*, *PTPN11*, A*SXL2*, and *WT1* remained stable or increased under SAIL treatment (Supplementary Table S7).

### Survival after selinexor and chemotherapy

Median survival time was 1.076 years in the included and 0.512 in the excluded patients (Supplementary Fig. S2). In univariate analysis, age (HR 0.129, 95%CI 0.025–0.666, *P* = 0.014) and type of AML (HR 0.222, 95%CI 0.055–0.906, *P* = 0.036) were predictive for OS (Supplementary Table S8). Variables considered for EFS in univariate analysis are shown in Supplementary Table S8. Mean overall survival (OS) was significantly longer in patients who underwent allogeneic hematopoietic cell transplantation (alloHCT, *P* = 0.014, Supplementary Fig. S4). OS was similar between patients with CR/CRi or no response (Supplementary Fig. S5) and patients with declining vs persisting clones (Supplementary Fig. S6). There was no significant difference in terms of OS comparing the cohort with molecular response versus all others.

### Selinexor maintenance treatment

**Fig. 2:**
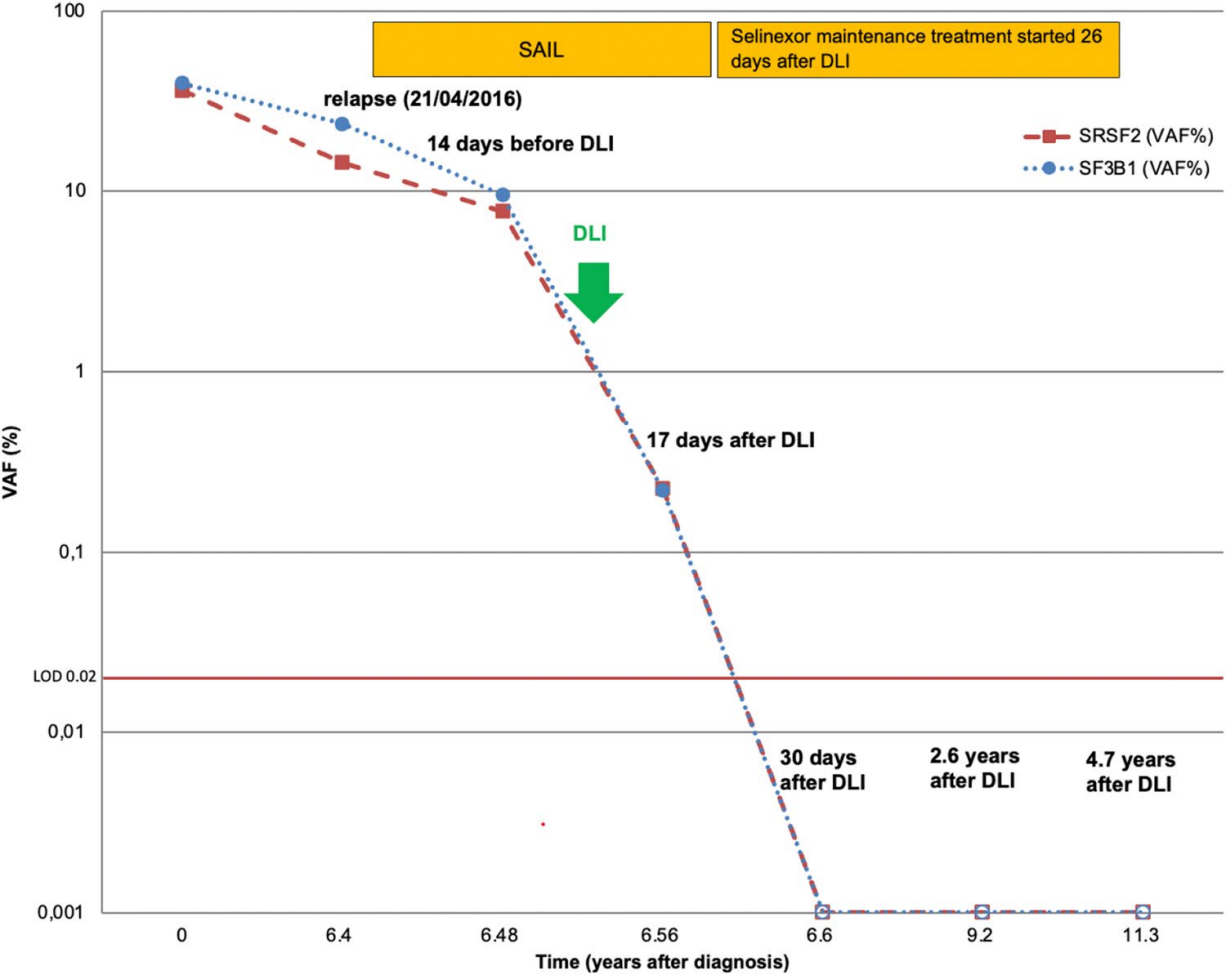
Molecular course of long-term patient under selinexor maintenance treatment. The x-axis marks the years after diagnosis. The orange and blue lines show the SRSF2 and SF3B1 MRD courses, respectively. The filled squares represent MRD positivity above the LOD of VAF 0.02% and the empty squares represent MRD negativity. Selinexor maintenance treatment started 26 days after DLI

One of the responding patients received selinexor as maintenance therapy for 4 years. The patient was diagnosed with de novo AML with normal cytogenetics, with *SF3B1* and *SRSF2* mutations. The patient initially received an HLA-identical transplant after myeloablative conditioning, but relapsed 6 years after alloHCT. One cycle of selinexor/chemotherapy was administered and the patient achieved CR. The patient continued selinexor maintenance treatment with 60-mg selinexor twice a week. *SF3B1* and *SRSF2* mutations were still present at the time of relapse and declined under SAIL treatment (Fig. 2). The patient received one course of donor lymphocyte infusion (DLI) (1 × 10^7^ CD3^+^ cells), which was tolerated well without signs of GvHD. MRD remained detectable 17 days after DLI. At 30 days after DLI treatment, both MRD markers turned negative under continued selinexor treatment. Under selinexor maintenance treatment, MRD remained negative until last follow-up at 4.9 years after SAIL treatment. The patient tolerated selinexor well with short-term nausea and dysgeusia after selinexor intake. Selinexor maintenance treatment was stopped 4 years after SAIL treatment and the patient remains in CR 14 months after the end of maintenance.

## Discussion

We analyzed the molecular profile of patients treated within the SAIL trial at initial diagnosis, at the time of screening for SAIL when the patient had relapsed or was refractory, and after the first cycle of SAIL induction chemotherapy. We evaluated subclonal response patterns and found that clones with mutations in *FLT3*, *SF3B1*, and *TP53* declined under SAIL treatment, whereas clones with mutations in *CUX1*, *GATA2*, *TET2*, *BCOR*, *DNMT3A*, *RAD21*, *ASXL1*, *SRSF2*, *RUNX1*, *NPM1*, *PTPN11*, A*SXL2*, and *WT1* remained stable or increased under SAIL treatment. Zhang et al. also found an association between *FLT3* mutation status and response in patients treated with selinexor when combined with the multikinase-inhibitor sorafenib [13]. *WT1* remained stable under SAIL treatment, whereas Wang et al. showed *WT1* as a reliable marker for response and relapse in AML patients treated with selinexor in combination with high-dose cytarabine and mitoxantrone [14]. *NPM1* was described before as a stable marker under selinexor [15]. The surprising effect on *TP53* may be explained by the mechanism of selinexor as an exportin inhibitor [16]. *ASXL1* and *SRSF2* were among the genes associated with molecular persistence. They are known as co-occurring genes associated with a dismal prognosis, which is concordant with our findings [17]. Although subclonal response patterns are interesting, they did not result in improved survival. Selinexor monotherapy in older relapsed/refractory AML patients with *TP53* mutations resulted in similar survival as in patients treated with physician’s choice [18].

We found a significant benefit in OS in patients undergoing alloHCT, confirming that alloHCT is an effective consolidation treatment after selinexor/chemotherapy.

One patient had a late relapse 6.4 years after alloHCT and received one cycle of SAIL treatment. He achieved CR and continued selinexor as a maintenance treatment. He was treated with DLI from the original donor and turned MRD negative 17 days after DLI and selinexor treatment and remained negative until 4.9 years after relapse. As the patient did not develop any signs of GvHD, it is not clear how much DLI or selinexor contributed to this long-term remission. Mutations occurred in *SRSF2* and *SF3B1* gene which are two spliceosome mutations leading to missense mutations. These are hotspot mutations (*SRSF2* NM_003016.4:c.284C > T,p.Pro95Leu, *SF3B1* NM_012433.2:c.1998G > T,p.Lys666Asn;) with different VAFs (*SRSF2* VAF 12.5%, *SF3B1* VAF 23.7%), suggesting that they occur either in different clones or have non-redundant pathogenic function. Spliceosome mutations are usually mutually exclusive, yet combined occurrence does occur and has been described before [19–21]. Another interesting long-term course was observed by Walker et al. [22] The patient was treated with selinexor monotherapy in the SOPRA trial for 40 months. Sequencing identified mutations in *IDH2*, *DNMT3A*, and *BCORL1* at the time before treatment and declining VAFs under selinexor. Monotherapy of selinexor in relapsed/refractory unfit AML patients was evaluated in the SOPRA trial and compared against physician’s choice [18]. CR/CRi was achieved in 11.9% vs 3.5%, respectively, with median OS of 3.2 vs 5.6 months, respectively [18]. Response to selinexor monotherapy was associated with six master regulator proteins, with five proteins having higher activity in responders (PKIA, ZDBF2, BCL11B, FHIT, and CAMK4), and one protein having lower activity in responders (MGST2) (18).

Our study is clearly limited by the small patient number, the low number of patients within each genetic subgroup, and some cases with incomplete data. Our study is therefore exploratory and the therapeutic effect of selinexor cannot be separated from the effect of chemotherapy or DLI/alloHCT.

In summary, we correlate response to genetic characteristics in a subset of the patients treated within the SAIL study, identify subclonal response patterns, and describe the effect and tolerability of selinexor long-term treatment in a patient with relapsed AML.

## Supplementary Information

Below is the link to the electronic supplementary material.

Supplementary file1 (PDF 573 KB)
