# Supplementary material for: Molecular response patterns in relapsed/refractory AML patients treated with selinexor and chemotherapy
Source: Ann Hematol. 2022 Dec 28;102(2):323–8. doi: 10.1007/s00277-022-05075-4 (PMC9889470; doi:10.1007/s00277-022-05075-4)

Molecular response patterns in relapsed/refractory AML patients treated with selinexor and chemotherapy

Supplementary information

Supplementary Tables

Supplementary Table S1. Genes included in our custom myeloid panel.

Gene	Exons	Gene	Exons	Gene	Exons
ASXL1	12	*GATA2*	2-6	*RUNX1*	complete
ASXL2	11+12	*IDH1*	4	*SETBP1*	4
BCOR	complete	*IDH2*	4	*SF3B1*	13-16
BCORL1	complete	*JAK2*	12, 14	*SMC1A*	2, 11, 16, 17
BRAF	Exon15	*KDM6A*	complete	*SMC3*	10, 13, 19, 23, 25, 28
CALR	9	*KIT*	2, 8-11, 13, 17	*SRSF2*	1
CBL	8, 9	*KRAS*	2-5	*STAG1*	complete
CEBPA	complete	*MPL*	10	*STAG2*	complete
CSF3R	14-17	*MYC*	2	*TET2*	3-11
CSNK1A1	3, 4	*NF1*	complete	*TP53*	2-11
DDX41	complete	*NPM1*	12	*U2AF1*	2, 6
DNMT3A	complete	*NRAS*	2-5	*WT1*	7, 9
ETNK1	3	*PHF6*	complete	*ZBTB7A*	2, 3
ETV6	complete	*PPM1D*	1-6	*ZRSR2*	complete
EZH2	complete	*PTPN11*	3, 13		
FLT3	14-16, 20	*RAD21*	complete		

Supplementary Table S2. Primer sequences used for NGS MRD analysis.

Primer name	Primer sequence
SF3B1_NGS_648F	GGTAAACACAAGGGCACTGGNNNNNNNNNNNNNNNNNGCATTCCTTCTTTATTGCCCTTC
SF3B1_NGS_648R	CGGACTACAGCTCCCATCATNNNNNNNNNNNNNNNNNACTTCTAAGATGTGGCAAGATGG
SRSF2_NGS_69_120LF	GGTAAACACAAGGGCACTGGNNNNNNNNTGAGGACGCTATGGATGCC
SRSF2_NGS_69_120LR	CGGACTACAGCTCCCATCATNNNNNNNNcctcagccccgtttacCTG

Supplementary Table S3. Comparison of patient demographics and baseline characteristics of included and excluded patients.

Characteristic	Included patients (n=15)	Excluded patients (n=27)	P
Age			**0.019**
Median (years)	49.1	59.6	
Range (years)	29-72	35-78	
Sex			0.78
Male - no. (%)	9 (60)	15 (56)	
Female - no. (%)	6 (40)	12 (44)	
ECOG performance status at diagnosis			0.48
0 - no. (%)	10 (66)	4 (15)	
1 - no. (%)	3 (20)	0 (0)	
2 - no. (%)	1 (7)	0 (0)	
Missing data - no. (%)	1 (7)	23 (85)	
ECOG performance status			0.49
0 - no. (%)	6 (40)	8 (30)	
1 - no. (%)	5 (33)	12 (44)	
2 - no. (%)	1 (7)	1 (4)	
3 - no. (%)	1 (7)	0 (0)	
Missing data - no. (%)	2 (13)	6 (22)	
Type of AML			0.37
De novo - no. (%)	11 (73)	19 (70)	

Secondary* - no. (%)	4 (27)	5 (19)	
Therapy-related -no. (%)	0 (0)	3 (11)	
Cytogenetic risk group			0.874
Favorable/intermediate – no. (%)	9 (60)	16 (59)	
Adverse – no. (%)	4 (27)	8 (30)	
missing data - no. (%)	2 (13)	3 (11)	
ELN at SAIL screening (9)			0.571
Favorable/intermediate – no. (%)	5 (33)	3 (11)	
Unfavorable – no. (%)	7 (47)	7 (26)	
Missing data – no. (%)	3 (20)	17 (63)	
WBC count			0.38
Median - (x10^9/l)	4.17	8.53	
Range - (x10^9/l)	0.6-12.9	0.4-85.0	
Hemoglobin			0.075
Median – g/dL	9.21	10.22	
Range – g/dL	7.1-12.5	8.3-13.4	
Platelet count			**0.027**
Median - (x10^9/l)	46.64	101.38	
Range - (x10^9/l)	10-98	3-346	
Prior HCT			0.307
Yes – no. (%)	8 (53)	10 (37)	
No – no. (%)	7 (47)	17 (63)	
aGVHD in patients receiving an allograft or DLIs (n=12; n=7)			0.4
Yes – no. (%)	8 (73)	2 (7)	
No – no. (%)	3 (27)	0 (0)	
Missing data - no. (%)	0 (0)	25 (93)	
cGVHD in transplanted receiving an allograft or DLIs (n=12; n=7)			0.4
Yes – no. (%)	3 (20)	0 (0)	
No – no. (%)	8 (53)	2 (7)	
Missing data - no. (%)	4 (27)	25 (93)	
Treatment after SAIL			0.011

Yes – no. (%)	15 (100)	9 (33)	
No – no. (%)	0 (0)	5 (19)	
Missing data - no. (%)	0 (0)	13 (48)	
Type of treatment after SAIL			0.077
alloHCT- no. (%)**	9 (60)	7 (26)	
DLI - no. (%)	3 (20)	2 (7)	
Intensive Chemotherapy - no. (%)	2 (13)	0 (0)	
Non-intensive chemotherapy - no. (%)	1 (7)	0 (0)	
No treatment - no. (%)	0 (0)	5 (19)	
Missing data - no. (%)	0 (0)	13 (48)	

Abbreviations: ECOG, performance status of the Eastern Cooperative Oncology Group; ELN, European LeukemiaNet; WBC, white blood cell count; HCT, hematopoietic stem cell transplantation; alloHCT, allogeneic hematopoietic stem cell transplantation; aGvHD, acute graft-versus-host disease; cGvHD, chronic graft-versus-host disease; DLI, donor lymphocyte infusion; P, P-value from two-sided chi-squared tests for categorical variables and from two-sided Student's T or Kolmogorov-Smirnov tests for continuous variables.

* secondary AML developing after an antecedent hematologic disorder;

** three patients received a second alloHCT.

Supplementary Table S4. Patient demographics and baseline characteristics (n=15).

Characteristic	Included patients (n=15)
Age	
Median (years)	49.1
Range (years)	29-72
Sex	
Male - no. (%)	9 (60)
Female - no. (%)	6 (40)
ECOG performance status at diagnosis	
0 - no. (%)	10 (66)
1 - no. (%)	3 (20)
2 - no. (%)	1 (7)
Missing data - no. (%)	1 (7)
ECOG performance status	
0 - no. (%)	6 (40)
1 - no. (%)	5 (33)
2 - no. (%)	1 (7)
3 - no. (%)	1 (7)
Missing data - no. (%)	2 (13)
Type of AML	
De novo - no. (%)	11 (73)
Secondary* - no. (%)	4 (27)
Therapy-related -no. (%)	0 (0)
Cytogenetic risk group	
Favorable/intermediate – no. (%)	9 (60)
Adverse – no. (%)	4 (27)
missing data - no. (%)	2 (13)
ELN at SAIL screening (9)	
Favorable/intermediate – no. (%)	5 (33)
Unfavorable – no. (%)	7 (47)
Missing data – no. (%)	3 (20)
WBC count	
Median - ($x10^9/l$)	4.17
Range - ($x10^9/l$)	0.6-12.9
Hemoglobin	
Median – g/dL	9.21
Range – g/dL	7.1-12.5

Platelet count	
Median - (x10^9/l)	46.64
Range - (x10^9/l)	10-98
Prior HCT	
Yes – no. (%)	8 (53)
No – no. (%)	7 (47)
aGvHD in patients receiving an allograft or DLIs (n=12)	
Yes – no. (%)	8 (73)
No – no. (%)	3 (27)
cGvHD in transplanted receiving an allograft or DLIs (n=12)	
Yes – no. (%)	3 (27)
No – no. (%)	8 (73)
Treatment after SAIL	
Yes – no. (%)	15 (100)
No – no. (%)	0 (0)
Type of treatment after SAIL	
alloHCT- no. (%)	9 (60)
DLI - no. (%)	3 (20)
Intensive chemotherapy - no. (%)	2 (13)
Non-intensive chemotherapy - no. (%)	1 (7)
No treatment - no. (%)	0 (0)
Missing data - no. (%)	0 (0)

Abbreviations: ECOG, performance status of the Eastern Cooperative Oncology Group; ELN, European LeukemiaNet; WBC, white blood cell count; HCT, hematopoietic stem cell transplantation; alloHCT, allogeneic hematopoietic stem cell transplantation; aGvHD, acute graft-versus-host disease; cGvHD, chronic graft-versus-host disease; DLI, donor lymphocyte infusion; P, P-value from two-sided chi-squared tests for categorical variables and from two-sided Student's T or Kolmogorov-Smirnov tests for continuous variables.

* secondary AML developing after an antecedent hematologic disorder;

** three patients received a second alloHCT.

Supplementary Table S5. Comparison of clinical characteristics between CR/CRi (n=7) and all other patients (n=8).

Characteristic	All (n=15)	CR/CRi after induction chemotherapy n=7	All others (MLFS, SD, PR, PD, after induction) n=8	P
Age				0.586
Median (years)	49.1	46.7	51.3	
Range (years)	29-72	34-72	29-69	
Sex				0.398
Male - no. (%)	9 (60)	5 (71)	4 (50)	
Female - no. (%)	6 (40)	2 (29)	4 (50)	
ECOG performance status at diagnosis				0.061
0 - no. (%)	10 (66)	7 (100)	3 (38)	
1 - no. (%)	3 (20)	0 (0)	3 (38)	
2 - no. (%)	1 (7)	0 (0)	1 (13)	
Missing data - no. (%)	1 (7)	0 (0)	1 (13)	
ECOG performance status				0.545
0 - no. (%)	6 (40)	3 (43)	3 (38)	
1 - no. (%)	5 (33)	2 (29)	3 (38)	
2 - no. (%)	1 (7)	0 (0)	1 (13)	
3 - no. (%)	1 (7)	1 (14)	0 (0)	
Missing data - no. (%)	2 (13)	1 (14)	1 (13)	
Type of AML				0.31
De novo - no. (%)	11 (73)	6 (86)	5 (62)	
Secondary* - no. (%)	4 (27)	1 (14)	3 (38)	
Therapy-induced -no. (%)	0 (0)	0 (0)	0 (0)	
Cytogenetic risk group				0.853
Favorable/intermediate – no. (%)	9 (60)	5 (71)	4 (50)	
Adverse – no. (%)	4 (27)	2 (29)	2 (25)	
Missing data - no. (%)	2 (13)	0 (0)	2 (25)	
ELN at SAIL screening (9)				0.079
Favorable/intermediate – no. (%)	5 (33)	4 (50)	1 (12)	

Unfavorable – no. (%)	7 (47)	2 (25)	5 (63)	
Missing data – no. (%)	3 (20)	2 (25)	2 (25)	
WBC count				0.54
Median - (x10^9/l)	4.17	3.43	4.91	
Range - (x10^9/l)	0.6-12.9	0.6-12.6	0.7-12.9	
Hemoglobin				0.664
Median – g/dL	9.21	9.41	9.01	
Range – g/dL	7.1-12.5	7.1-12.5	7.3-11.0	
Platelet count				0.443
Median - (x10^9/l)	46.64	52.71	40.57	
Range - (x10^9/l)	10-98	15-98	10-86	
Prior HCT				0.782
Yes – no. (%)	8 (53)	4 (57)	4 (50)	
No – no. (%)	7 (47)	3 (43)	4 (50)	
AlloHCT after SAIL				0.833
Yes – no. (%)	9 (60)	4 (57)	5 (63)	
No – no. (%)	6 (40)	3 (43)	3 (37)	
aGVHD in patients receiving an allograft or DLIs (n=12)				0.387
Yes – no. (%)	8 (73)	5 (83)	3 (60)	
No – no. (%)	3 (27)	1 (17)	2 (40)	
cGVHD in transplanted receiving an allograft or DLIs (n=12)				0.621
Yes – no. (%)	3 (27)	2 (33)	1 (20)	
No – no. (%)	8 (73)	4 (67)	4 (80)	
Treatment after SAIL				n.a.
Yes – no. (%)	15 (100)	7 (100)	8 (100)	
No – no. (%)	0 (0)	0 (0)	0 (0)	
Type of treatment after SAIL				0.335
AlloHCT- no. (%)	9 (60)	4 (57)	5 (62)	
DLI - no. (%)	3 (20)	2 (29)	1 (12)	
Intensive chemotherapy - no. (%)	2 (13)	0 (0)	2 (26)	
Non-intensive chemotherapy - no. (%)	1 (7)	1 (14)	0 (0)	
No treatment - no. (%)	0 (0)	0 (0)	0 (0)	

Abbreviations: CR, complete remission; CRi, complete remission with incomplete hematologic recovery; MLFS, morphologic leukemia-free state; SD, stable disease; PR, partial remission; PD, progressive disease; ECOG, performance status of the Eastern Cooperative Oncology Group; ELN, European LeukemiaNet; WBC, white blood cell count; HCT, hematopoietic stem cell transplantation; alloHCT, allogeneic hematopoietic stem cell transplantation; aGvHD, acute graft-versus-host disease; cGvHD, chronic graft-versus-host disease; DLI, donor lymphocyte infusion; P, P-value from two-sided chi-squared tests for categorical variables and from two-sided Student's T or Kolmogorov-Smirnov tests for continuous variables.

* secondary AML developing after an antecedent hematologic disorder;

Supplementary Table S6. Comparison of molecular characteristics between CR/CRi (n=8) and all other patients (n=10).

Muation class	Mutated gene	All (n=15)	CR/Cri after inducti on n=7	All others (SD, PR, PD, MLFS, after induction) n=8	P
Epigenetic modifiers	*ASXL1*				0.605
	Mutated – no. (%)	3 (20)	1 (33)	2 (67)	
	Wildtype – no. (%)	12 (80)	6 (50)	6 (50)	
	ASXL2				0.038
	Mutated – no. (%)	3 (20)	3 (100)	0 (0)	
	Wildtype – no. (%)	12 (80)	4 (33)	8 (67)	
	DNMT3A				0.605
	Mutated – no. (%)	3 (20)	1 (33)	2 (67)	
	Wildtype – no. (%)	12 (80)	6 (50)	6 (50)	
	IDH2				0.333
	Mutated – no. (%)	1 (7)	0 (0)	1 (100)	
	Wildtype – no. (%)	14 (93)	7 (50)	7 (50)	
	KDM6A				0.333
	Mutated – no. (%)	1 (7)	0 (0)	1 (100)	
	Wildtype – no. (%)	14 (93)	7 (50)	7 (50)	
	TET2				0.605

	Mutated – no. (%)	3 (20)	1 (33)	2 (67)	
	Wildtype – no. (%)	12 (80)	6 (50)	6 (50)	
**Nucleo-phosmin**	*NPM1*– no. (%)				0.919
	Mutated – no. (%)	2 (13)	1 (50)	1 (50)	
	Wildtype – no. (%)	13 (87)	6 (46)	7 (54)	
**Cohesin complex**	*RAD21*				n.a.
	Mutated – no. (%)	0 (0)	0 (0)	0 (0)	
	Wildtype – no. (%)	15 (100)	7 (100)	8 (100)	
	SMC1A				0.333
	Mutated – no. (%)	1 (7)	0 (0)	1 (100)	
	Wildtype – no. (%)	14 (93)	7 (50)	7 (50)	
**Signal transduction**	*CBL*				0.333
	Mutated – no. (%)	1 (7)	0 (0)	1 (100)	
	Wildtype – no. (%)	14 (93)	7 (50)	7 (50)	
	FLT3 – no. (%)				0.104
	Mutated – no. (%)	2 (13)	2 (100)	0 (0)	
	Wildtype – no. (%)	13 (87)	5 (38)	8 (62)	
	KRAS				0.333
	Mutated – no. (%)	1 (7)	0 (0)	1 (100)	
	Wildtype – no. (%)	14 (93)	7 (50)	7 (50)	
	NF1				0.333
	Mutated – no. (%)	1 (7)	0 (0)	1 (100)	
	Wildtype – no. (%)	14 (93)	7 (50)	7 (50)	
	PTPN11				0.919
	Mutated – no. (%)	2 (13)	1 (50)	1 (50)	
	Wildtype – no. (%)	13 (87)	6 (46)	7 (54)	
**Spliceosome**	*SF3B1*				0.919
	Mutated – no. (%)	2 (13)	1 (50)	1 (50)	
	Wildtype – no. (%)	13 (87)	6 (46)	7 (54)	
	SRSF2				0.605
	Mutated – no. (%)	3 (20)	1 (33)	2 (67)	
	Wildtype – no. (%)	12 (80)	6 (50)	6 (50)	
	U2AF1				0.333
	Mutated – no. (%)	1 (7)	0 ()	1 (100)	
	Wildtype – no. (%)	14 (93)	7 (50)	7 (50)	
	ZBTB7A				0.268
	Mutated – no. (%)	1 (7)	1 (100)	0 (0)	
	Wildtype – no. (%)	14 (93)	6 (43)	8 (57)	
**Myeloid TF‘s**	*BCOR*				0.333
	Mutated – no. (%)	1 (7)	0 (0)	1 (100)	
	Wildtype – no. (%)	14 (93)	7 (50)	7 (50)	

	CEBPA				0.333
	Mutated – no. (%)	1 (7)	0 (0)	1 (100)	
	Wildtype – no. (%)	14 (93)	7 (50)	7 (50)	
	CUX1				0.070
	Mutated – no. (%)	3 (20)	0 (0)	3 (100)	
	Wildtype – no. (%)	12 (80)	7 (58)	5 (42)	
	GATA2				0.919
	Mutated – no. (%)	2 (13)	1 (50)	1 (50)	
	Wildtype – no. (%)	13 (87)	6 (46)	7 (54)	
	RUNX1				0.31
	Mutated – no. (%)	4 (27)	1 (25)	3 (75)	
	Wildtype – no. (%)	11 (73)	6 (55)	5 (45)	
	SETBP1				0.333
	Mutated – no. (%)	1 (7)	0 (0)	1 (100)	
	Wildtype – no. (%)	14 (93)	7 (50)	7 (50)	
**Tumor suppressors**	*PPM1D*				0.333
	Mutated – no. (%)	1 (7)	0 (0)	1 (100)	
	Wildtype – no. (%)	14 (93)	7 (50)	7 (50)	
	TP53				0.104
	Mutated – no. (%)	2 (13)	2 (100)	0 (0)	
	Wildtype – no. (%)	13 (87)	5 (38)	8 (62)	
	WT1				0.155
	Mutated – no. (%)	2 (13)	0 (0)	2 (100)	
	Wildtype – no. (%)	13 (87)	7 (54)	6 (46)	

Abbreviations: CR, complete remission; CRi, complete remission with incomplete hematologic recovery; MLFS, morphologic leukemia-free state; SD, stable disease; PR, partial remission; PD, progressive disease; P, P-value from two-sided chi-squared tests for categorical variables; TF, transcription factor.

Supplementary Table S7. Response patterns of gene mutations under SAIL treatment.

Gene	Molecular Response association	Based on number of patients (n)
RUNX1	Molecular non-response	4
GATA2	Molecular non-response	2
TET2	Molecular non-response	3
ASXL1	Molecular non-response	3
ASXL2	Molecular non-response	3
BCOR	Molecular non-response	1
CUX1	Molecular non-response	3
DNMT3A	Molecular non-response	3
FLT3	Molecular response	2
NPM1	Molecular non-response	2
PTPN11	Molecular non-response	2
SRSF2	Molecular non-response	3
TP53	Molecular response	2
SF3B1	Molecular response	2
WT1	Molecular non-response	2

Note: Molecular response was defined as VAF negativity in the follow-up sample after SAIL treatment in comparison to the relapse sample.

Supplementary Table S8. Univariate Analysis for OS and EFS in 15 SAIL patients.

Endpoint	Variables in the model	Univariate analysis		
		HR	95% CI	P
OS	Age <median vs >median	0.129	0.025-0.666	**0.014**
	Male vs. female	0.513	0.144-1.832	0.304
	ECOG at SAIL screening 0 or 1 vs. 2	0.500	0.100-2.496	0.398

	alloHCT after SAIL yes vs. no	0.331	0.094-1.172	0.087
	Type of AML primary vs. secondary/therapy-induced	0.222	0.055-0.906	**0.036**
	Hemoglobin (g/dL) <median vs. >median	2.096	0.580-7.571	0.259
	WBC count (x10^9/l) <median vs. >median	0.713	0.201-2.529	0.601
	Platelet count (x10^9/l) <median vs. >median	1.828	0.504-6.635	0.359
	FLT3 mut vs wt	0.770	0.096-6.184	0.805
	ASXL1 mut vs wt	0.770	0.162-3.661	0.743
	ASXL2 mut vs wt	0.727	0.150-3.528	0.693
	CUX1 mut vs wt	1.137	0.235-5.502	0.873
	DNMT3A mut vs wt	1.354	0.279-6.585	0.707
	GATA2 mut vs wt	0.519	0.064-4.189	0.538
	SRSF2 mut vs wt	0.964	0.204-4.564	0.963
	TET2 mut vs wt	3.881	0.914-16.470	0.066
	TP53 mut vs wt	4.351	0.787-24.066	0.092
EFS	Age <median vs >median	0.485	0.079-2.966	0.433
	Male vs. female	0.704	0.116-4.265	0.703
	ECOG at SAIL 0 and 1 vs. 2 and 3	0.289	0.018-4.648	0.381
	alloHCT after SAIL yes vs. no	0.134	0.012-1.507	0.104
	Type of AML primary vs. secondary/therapy-induced	1.122	0.122-10.328	0.919
	Hemoglobin (g/dL) <median vs. >median	3.934	0.422-36.690	0.229
	WBC count (x10^9/l) <median vs. >median	0.696	0.113-4.270	0.695
	Platelet count (x10^9/l) <median vs. >median	1.605	0.261-9.872	0.610
	FLT3 mut vs wt	2.179	0.302-15.721	0.440

	DNMT3A mut vs wt	0.296	0.031-2.827	0.290
	GATA2 mut vs wt	0.638	0.071-5.772	0.689
	TP53 mut vs wt	1.420	0.234-8.597	0.703

Note: Genes were considered for univariate analysis if they were detectable in at least 3 patients. Hazard ratios greater than or less than 1 indicate an increased or decreased risk, respectively, of an event for the first category listed.

Patient number was too small for a useful calculation for the following variables: *BCOR, CBL, CEBPA, IHD2, KDM6A, KRAS, NF1, PPM1D, RAD21, RUNX1, SETBP1, SMC1A, TET2, U2AF1, ZBTB7A.*

Supplementary Figures

Supplementary Figure S1. Consort diagram of studied patients.

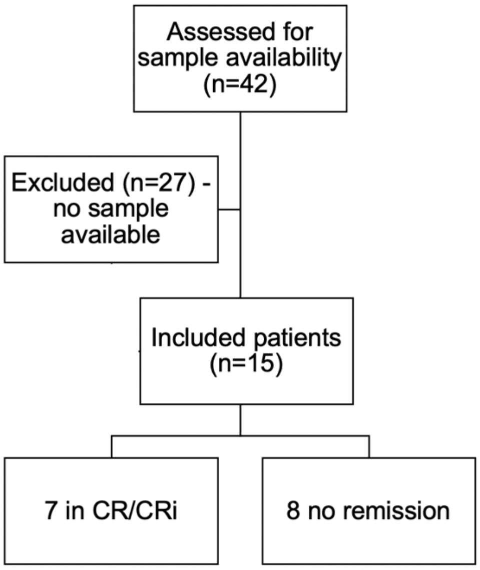

Supplementary Figure S2. Comparison of (A) EFS and (B) OS between included and excluded patients.

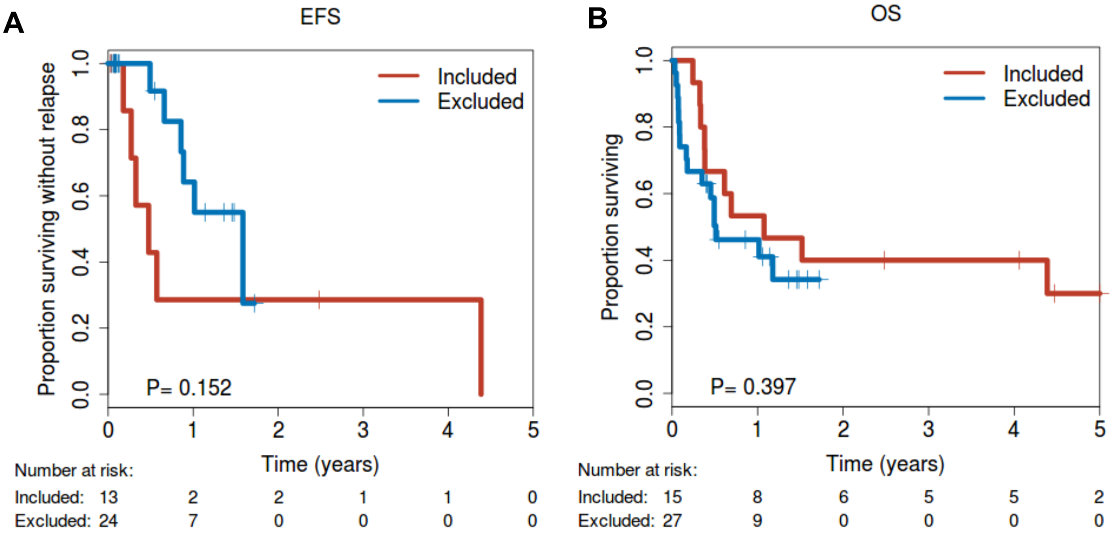

Supplementary Figure S3. Patient-specific clonal evolution from diagnosis to relapse/refractoriness and follow-up after cycle one of SAIL treatment.

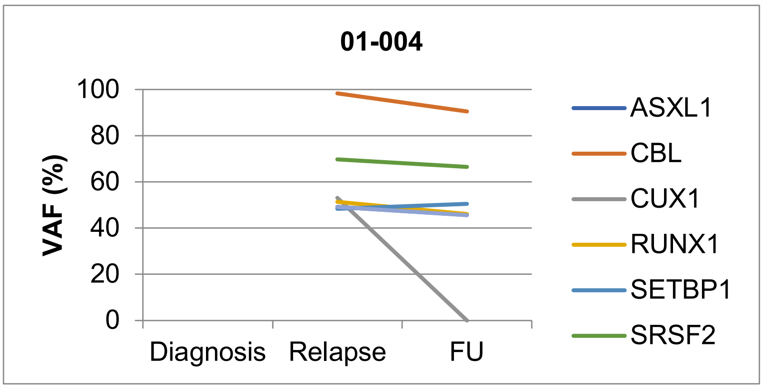

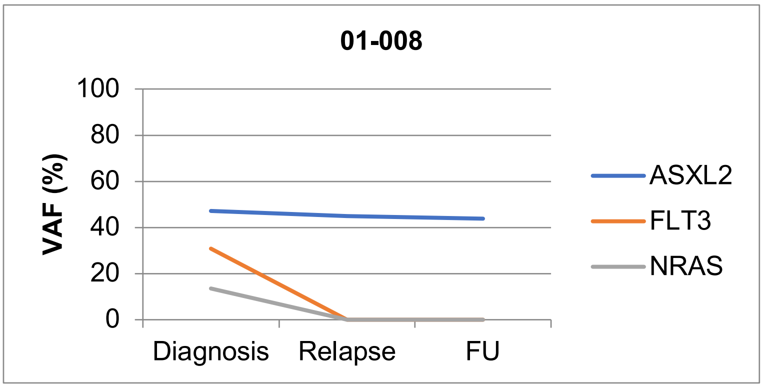
01-008
100
80
60
40
20
0
VAF (%)
Diagnosis
Relapse
FU
ASXL2
FLT3
NRAS

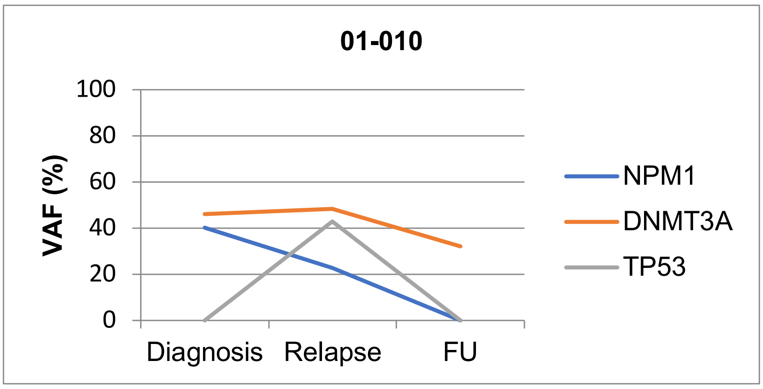
01-010
100
80
60
40
20
0
VAF (%)
Diagnosis
Relapse
FU
NPM1
DNMT3A
TP53

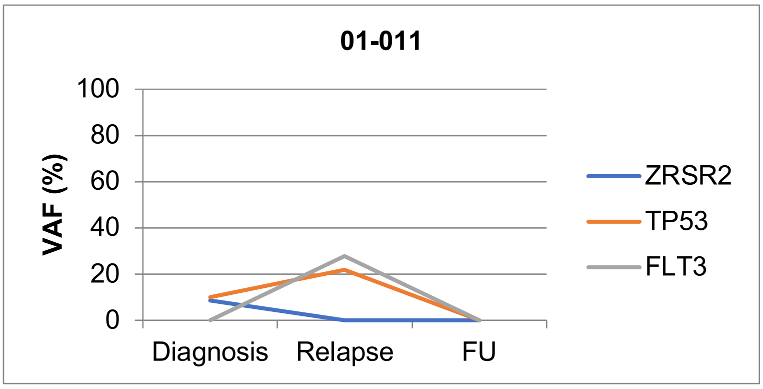
01-011
100
80
60
40
20
0
VAF (%)
Diagnosis
Relapse
FU
ZRSR2
TP53
FLT3

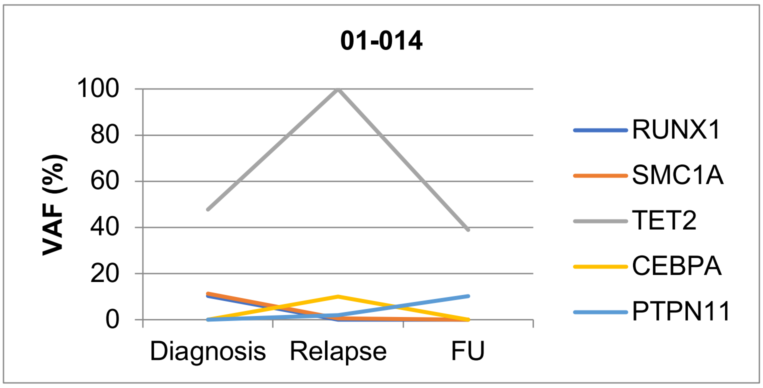
01-014
100
80
60
40
20
0
VAF (%)
Diagnosis
Relapse
FU
RUNX1
SMC1A
TET2
CEBPA
PTPN11

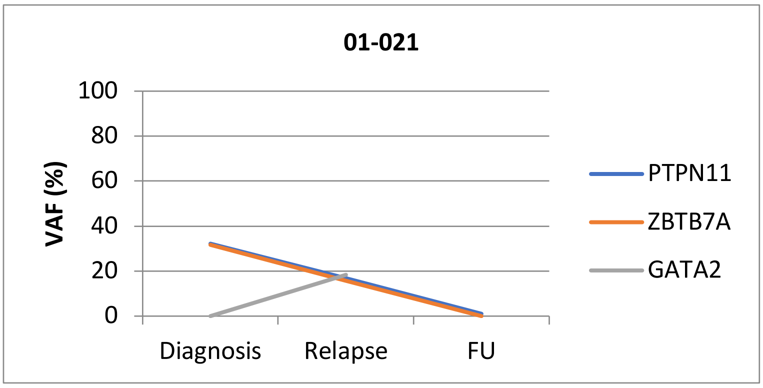
01-021
VAF (%)
100
80
60
40
20
0
Diagnosis
Relapse
FU
PTPN11
ZBTB7A
GATA2

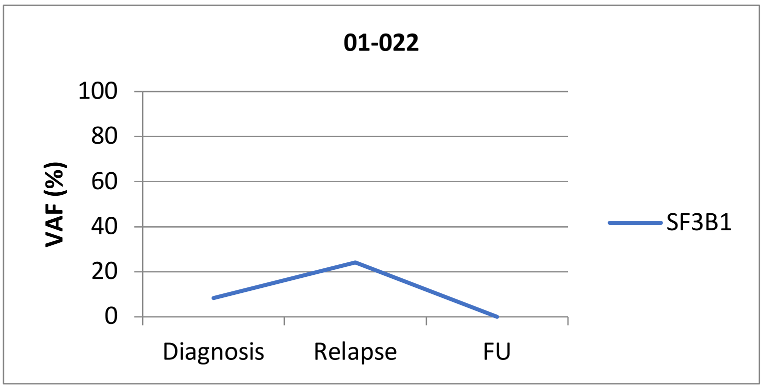
01-022
VAF (%)
100
80
60
40
20
0
Diagnosis
Relapse
FU
SF3B1

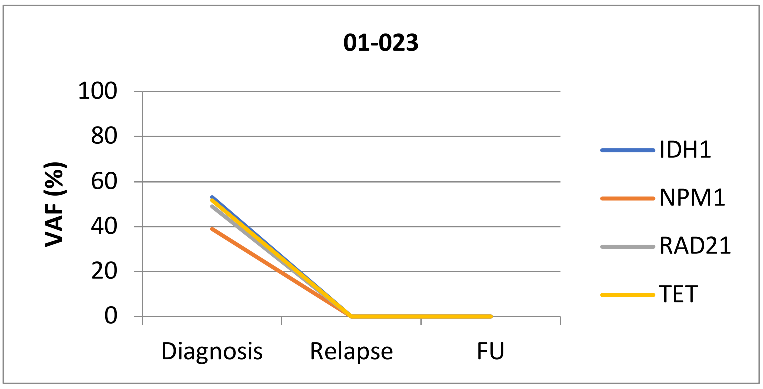
01-023
VAF (%)
100
80
60
40
20
0
Diagnosis
Relapse
FU
IDH1
NPM1
RAD21
TET

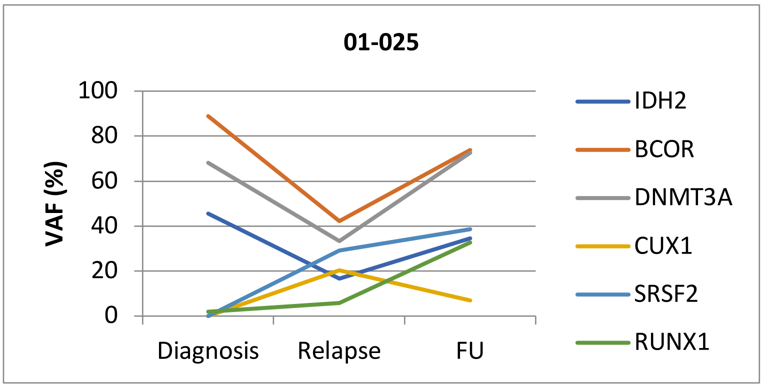
01-025
VAF (%)
100
80
60
40
20
0
Diagnosis
Relapse
FU
IDH2
BCOR
DNMT3A
CUX1
SRSF2
RUNX1

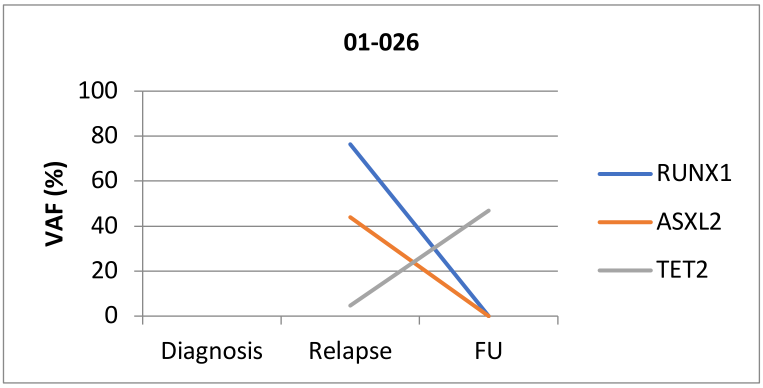
01-026
VAF (%)
100
80
60
40
20
0
Diagnosis
Relapse
FU
RUNX1
ASXL2
TET2

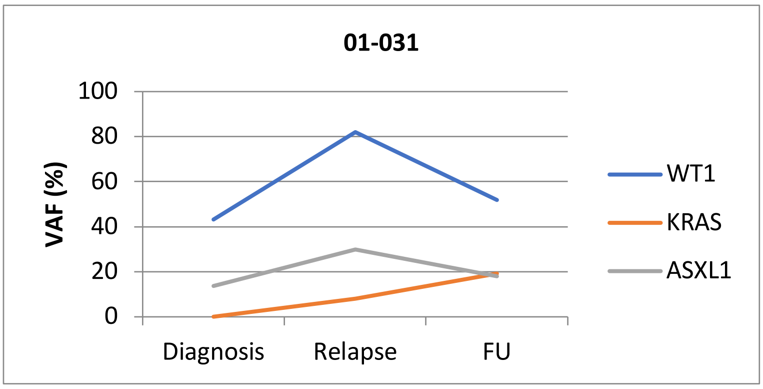
01-031
VAF (%)
100
80
60
40
20
0
Diagnosis
Relapse
FU
WT1
KRAS
ASXL1

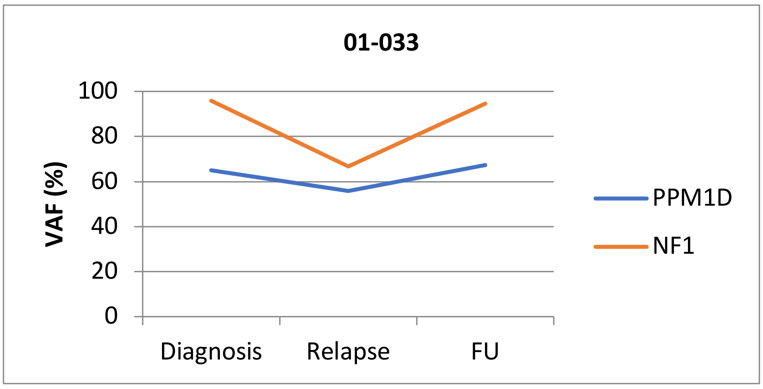
01-033
VAF (%)
100
80
60
40
20
0
Diagnosis
Relapse
FU
PPM1D
NF1

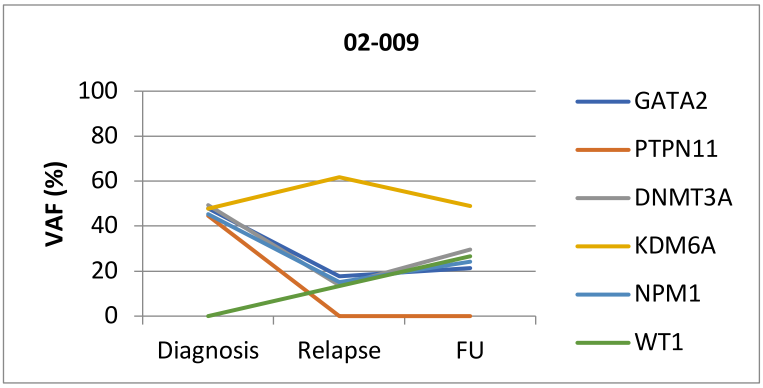
02-009
VAF (%)
100
80
60
40
20
0
Diagnosis
Relapse
FU
GATA2
PTPN11
DNMT3A
KDM6A
NPM1
WT1

Supplementary Figure S4. OS of patients undergoing alloHCT after SAIL induction vs all other patients.

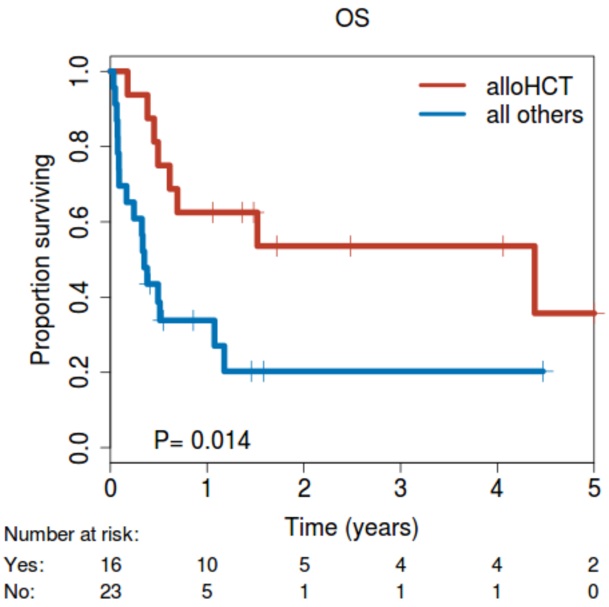

Supplementary Figure S5. OS in patients achieving CR/CRi vs. patients with other response to SAIL induction therapy.

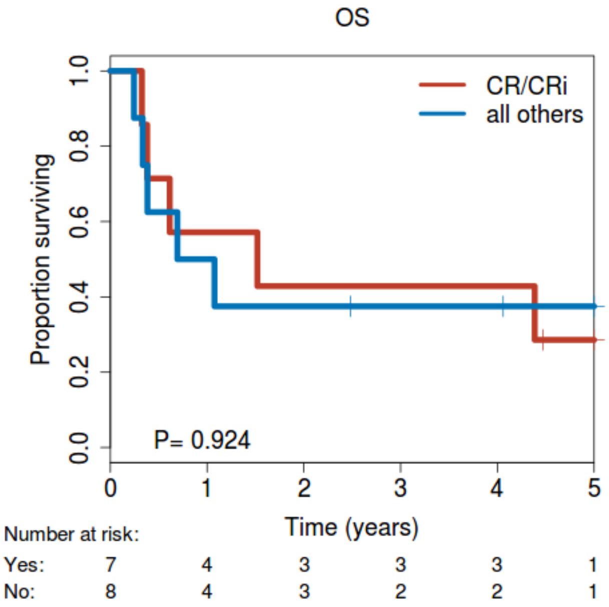

Supplementary Figure S6. OS in patients with declining and persisting clones after one cycle of SAIL treatment.

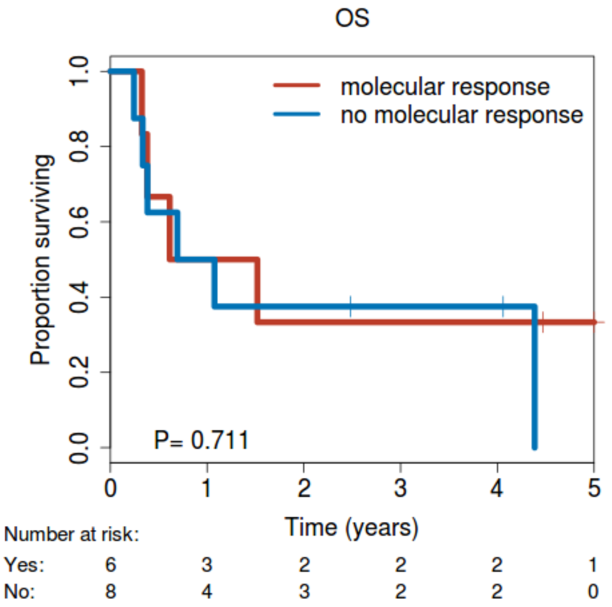

Supplement: Supplementary file 1 — Supplementary file1 (PDF 573 KB) [file 277_2022_5075_MOESM1_ESM.pdf]
